# Supplementary material for: GrgA as a potential target of selective antichlamydials
Source: PLoS One. 2019 Mar 1;14(3):e0212874. doi: 10.1371/journal.pone.0212874 (PMC6396966; doi:10.1371/journal.pone.0212874)
Supplement: S1 Table — (PDF) [file pone.0212874.s001.pdf]

S1 Table. Primers for amplifying and sequencing SNPs and resistance markers

Primer	Sequence (5’-3’)
rpoB F	CAATCCTCTTCAACATCAGC
rpoB R*	CGTTCGAGAAAGAATGAA
pdf F*	CTCCAGAATCAACACCGA
pdf R	GTGTTGGAGCATCTAGGG
16s rRNA F*	CTTGATGTGGATGGTCTC
16s rRNA R	CTTATTACCAGCTCGCC
SNP1 F	GTTGAGCTGCAAG GAAAC
SNP1 R*	TCTTAAAGGTAATTCTGCCG
SNP2 F*	CTCATCCCG GCTGAATC
SNP2 R	GATTCCATGACTCCCAGATC
SNP3 F*	CCGGAGTGGATGGT AGTC
SNP3 R	ACGCTGCCATCAGACACC
SNP4 F*	GGTCTAGTCC AACAGCC
SNP4 R	CTCGGAAAGATTGGAGAG

Asterisks signify primers for sequencing in addition to amplification.
