# Supplementary material for: GrgA as a potential target of selective antichlamydials
Source: PLoS One. 2019 Mar 1;14(3):e0212874. doi: 10.1371/journal.pone.0212874 (PMC6396966; doi:10.1371/journal.pone.0212874)
Supplement: S2 Table — (PDF) [file pone.0212874.s002.pdf]

S2 Table. Possible genotypes and actual available clonal populations obtained from MoPn_Rif^R X MCR_LBM^R recombination

Genotype	SNP1 MOMP	SNP2 Npt1	SNP3 TC0412	SNP4 GrgA	Clonal population
1	W	W	W	W	w1c1, w1c7, w1c15, w5c1, w5c2, w5c3
2	M	W	W	W	w1c6, w1c8, w1c13
3	W	M	W	W	Not available
4	W	W	M	W	w1c3
5	W	W	W	M	Not available
6	M	M	W	W	w1c4, w1c10, w1c11
7	M	W	M	W	Not available
8	M	W	W	M	Not available
9	W	M	M	W	w1c12, w2c2, w2c4, w2c5
10	W	M	W	M	Not available
11	W	W	M	M	w2c10
12	M	M	M	W	w1c2, w1c5, w1c9, w2c1, w2c3, w2c6, w3c1, w3c3, w3c4, w3c5
13	M	M	W	M	Not available
14	M	W	M	M	Not available
15	W	M	M	M	Not available
16	M	M	M	M	w2c7, w2c8, w3c2, w5c4
