# Supplementary material for: GrgA as a potential target of selective antichlamydials
Source: PLoS One. 2019 Mar 1;14(3):e0212874. doi: 10.1371/journal.pone.0212874 (PMC6396966; doi:10.1371/journal.pone.0212874)
Supplement: S3 Table — (PDF) [file pone.0212874.s003.pdf]

S3 Table. Possible genotypes and actual available clonal populations obtained from MoPn_Spc^R and MCR_Rif^R recombination

Genotype	SNP1 MOMP	SNP2 Npt1	SNP3 TC0412	SNP4 GrgA	Clonal population
1	W	W	W	W	r4s1, r4s2, r4s3, r4s4, r4s5, r4s6, r4s7, r4s8, r4s9, r4s10, r5s3, r5s4, r5s5, r5s6, r5s7
2	M	W	W	W	Not available
3	W	M	W	W	Not available
4	W	W	M	W	r4s11, r5s2,
5	W	W	W	M	r8s1, r8s2, r8s4, r8s6, r8s7, r8s9, r8s10, r8s11, r8s24
6	M	M	W	W	Not available
7	M	W	M	W	Not available
8	M	W	W	M	r5s1
9	W	M	M	W	Not available
10	W	M	W	M	Not available
11	W	W	M	M	r4s12, r4s13
12	M	M	M	W	Not available
13	M	M	W	M	Not available
14	M	W	M	M	r1s4, r8s5, r8s8, r8s21, r8s22
15	W	M	M	M	Not available
16	M	M	M	M	Not available
