# Supplementary material for: GrgA as a potential target of selective antichlamydials
Source: PLoS One. 2019 Mar 1;14(3):e0212874. doi: 10.1371/journal.pone.0212874 (PMC6396966; doi:10.1371/journal.pone.0212874)
Supplement: S4 Table — (PDF) [file pone.0212874.s004.pdf]

S4 Table. The S4(R51G GrgA) allele is both necessary and sufficient for BAH resistance

	SNP1 MOMP	SNP2 Npt1	SNP3 TC0412	SNP4 GrgA	Clonal population	Phenotype
S4 R51G GrgA	M	M	M	M	w3c2, w5c4	Resistant
	M	M	W	M	Not available	
	M	W	M	M	r1c4, r8s5	Resistant
	W	M	M	M	Not available	
	M	W	W	M	r5s1	Resistant
	W	M	W	M	Not available	
	W	W	M	M	w2c10, r4s12, r4s13	Resistant
	W	W	W	M	r8s6, r8s7, r8s11	Resistant
S4 wtGrgA	W	W	W	W	r4s9, r5s7, w1c15, w5c2	Susceptible
	W	W	M	W	w1c3, r4s11, r5s2	Susceptible
	W	M	W	W	Not available	
	M	W	W	W	w1c13	Susceptible
	W	M	M	W	w2c2, w1c12, w2c4	Susceptible
	M	W	M	W	Not available	
	M	M	W	W	w1c10, w1c4	Susceptible
	M	M	M	W	w3c4, w3c5	Susceptible

BAH inhibition phenotype (susceptible or resistant) of clonal populations arranged by the allele (wild-type or mutant, abbreviated as W or M, respectively) of the S4(GrgA) locus. Listed clonal populations tested for BAH susceptibility were from either MoPn_Rif^R X MCR_LBM^R recombination or MoPn_SpcR X MCR_Rif^R recombination.
