# Supplementary material for: GrgA as a potential target of selective antichlamydials
Source: PLoS One. 2019 Mar 1;14(3):e0212874. doi: 10.1371/journal.pone.0212874 (PMC6396966; doi:10.1371/journal.pone.0212874)
Supplement: S1 Fig — Chlamydiae grown without or with indicated concentrations of CF0001 were stained green using an anti-chlamydial LPS antibody 24 h post-inoculation. Host cells (red) were counter-stained with Evan blue. (PDF) [file pone.0212874.s005.pdf]

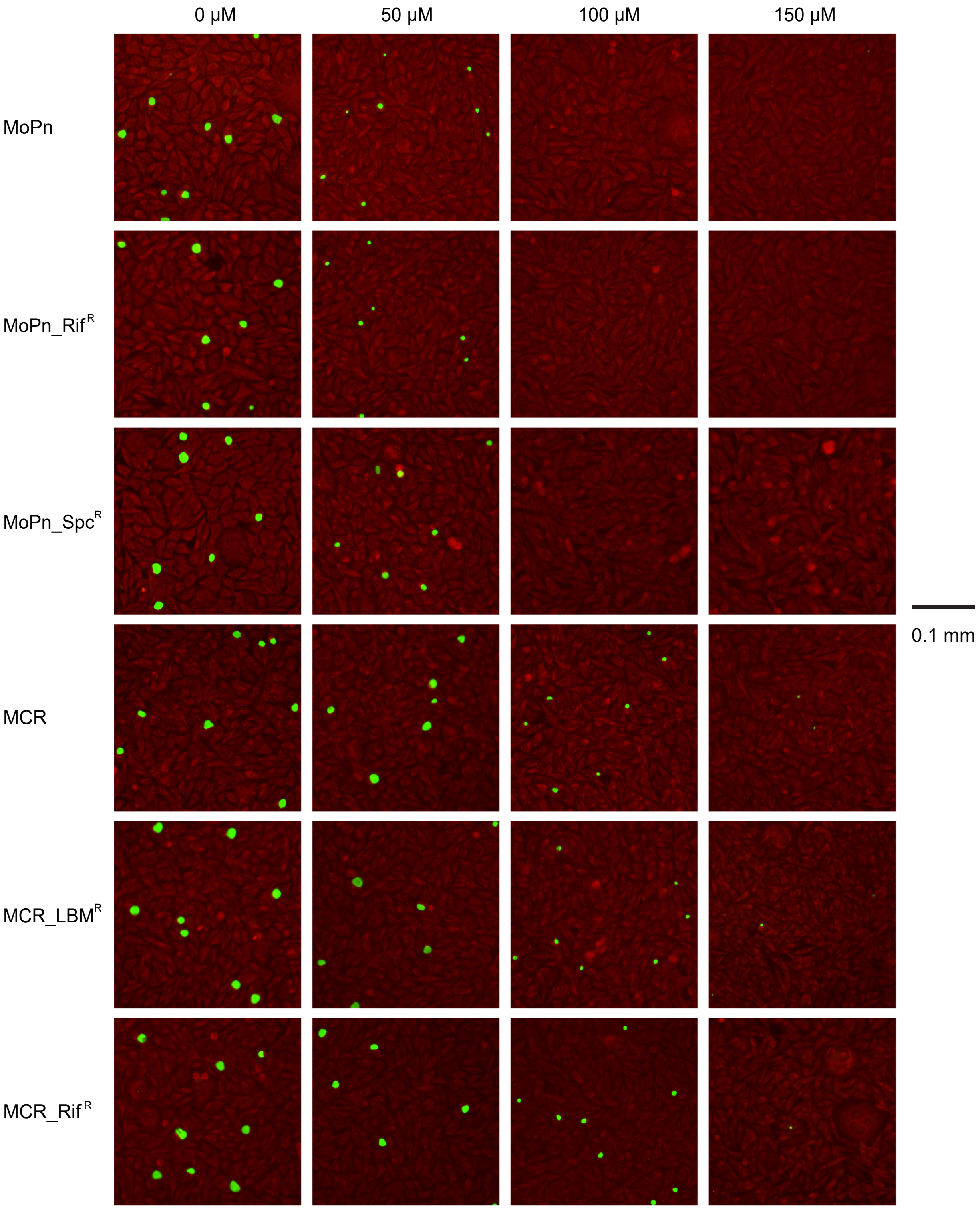

S1 Figure. Rifampin-, spectinomycin- and LBM415-resistance caused by mutations in rpoB, 16S rRNA and defA, respectively, in either MoPn or MCR do not change CF0001 inhibition efficiency. Chlamydiae grown without or with indicated concentrations of CF0001 were stained green using an anti-chlamydial LPS antibody 24 h post-inoculation. Host cells (red) were counter-stained with Evan blue.
